# Microstructure and Mechanical Properties of Vacuum Plasma Sprayed HfC, TiC, and HfC/TiC Ultra-High-Temperature Ceramic Coatings

**DOI:** 10.3390/ma13010124

**Published:** 2019-12-26

**Authors:** Ho Seok Kim, Bo Ram Kang, Seong Man Choi

**Affiliations:** 1 High-enthalpy Plasma Research Center, Jeonbuk National University, Wanju-gun 55317, Korea; hoseok@jbnu.ac.kr (H.S.K.); brkang@jbnu.ac.kr (B.R.K.); 2 Department of Aerospace Engineering, Jeonbuk National University, Jeonju 54896, Korea

**Keywords:** plasma spraying, vacuum plasma spraying, ultra-high-temperature ceramics, hafnium carbide, titanium carbide

## Abstract

To improve the oxidation resistance of carbon composites at high temperatures, hafnium carbide (HfC) and titanium carbide (TiC) ultra-high-temperature ceramic coatings were deposited using vacuum plasma spraying. Single-layer HfC and TiC coatings and multilayer HfC/TiC coatings were fabricated and compared. The microstructure and composition of the fabricated coatings were analyzed using field-emission scanning electron microscopy and energy dispersive X-ray spectroscopy. The coating thicknesses of the HfC and TiC single-layer coatings were 165 µm and 140 µm, respectively, while the thicknesses of the HfC and TiC layers in the HfC/TiC multi-layer coating were 40 µm and 50 µm, respectively. No oxides were observed in any of the coating layers. The porosity was analyzed from cross-sectional images of the coating layers obtained using optical microscopy. Five random areas for each coating layer specimen were analyzed, and average porosity values of approximately 16.8% for the HfC coating and 22.5% for the TiC coating were determined. Furthermore, the mechanical properties of the coating layers were investigated by measuring the hardness of the cross section and surface roughness. The hardness values of the HfC and TiC coatings were 1650.7 HV and 753.6 HV, respectively. The hardness values of the HfC and TiC layers in the multilayer sample were 1563.5 HV and 1059.2 HV, respectively. The roughness values were 5.71 µm for the HfC coating, 4.30 µm for the TiC coating, and 3.32 µm for the HfC/TiC coating.

## 1. Introduction

Ultra-high-temperature ceramics (UHTC) are materials of interest for extreme environments, such as those experienced for aerospace applications. UHTC materials have melting points above 3000 °C, very high hardness, and excellent chemical stability and strength at high temperatures [1]. Hafnium carbide (HfC) and titanium carbide (TiC) have melting points of 3958 °C and 3100 °C, respectively, and can be used as materials in thermal protection systems. HfC has a low oxygen diffusion coefficient and excellent thermal stability; it is an excellent heat-resistant material because the melting point of HfO_2_ is very high (2758 °C) [2,3,4]. TiC is resistant to thermal shock, has excellent wear resistance [5,6], and is suitable for plasma spray coating due to its relatively low melting point.

Plasma spray coatings can improve the abrasion resistance, corrosion resistance, and thermal resistance of a surface. The coating layer is formed by melting a precursor material, accelerating the melted droplets, and depositing them on a substrate using a high-temperature plasma. Plasma spray coating methods are generally divided into VPS and atmospheric plasma spraying (APS) processes. In the APS process, the coating layer is easily oxidized by oxygen in the air, and the density of the coating layer is low. In contrast, the VPS process uses a low-pressure argon atmosphere that inhibits oxidation of the coating layer, while the high velocity of the molten droplets facilitates the formation of highly dense coatings [7,8].

Carbon/carbon (C/C) composites are attractive materials for various applications due to advantages such as thermal shock resistance, high melting point, and excellent mechanical properties under high-temperature environments. However, as carbon-based materials oxidize in air at temperatures above 500 °C [9], UHTC coatings can be used to protect the C/C composites from thermal damage. In previous research, Yoo et al. successfully formed an HfC coating using VPS [10]. The formation of a single HfC coating layer using VPS is a unique result. A previous study used a chemical vapor reaction (CVR) process to deposit a SiC layer on the surface of a C/C composite to enable HfC coating using VPS. Yang studied an HfC-ZrC-SiC multiphase protective coating using supersonic atmospheric plasma spraying (SAPS) [11] and Zhang fabricated a ZrB_2_-SiC coating using SAPS [12]. Corral synthesized SiC and ZrB_2_ coatings using dip-coating [13]. Various coating methods have been developed for C/C composites protection, but HfC single-layer and multilayer coating studies using VPS have not been reported yet. The purpose of this study was to compare single-layer coatings of HfC or TiC and multilayer HfC/TiC coatings deposited on similar C/C substrates. In this work, we developed single layers of HfC and TiC and HfC/TiC multilayer coatings that can be applied to C/C composites as protective coatings using a VPS system. We analyzed the microstructure, composition, and porosity of the three different types of coatings. In addition, the hardness of the cross sections of the layers and the surface roughness of the samples were measured.

## 2. Experimental Details 

**Figure 1:**
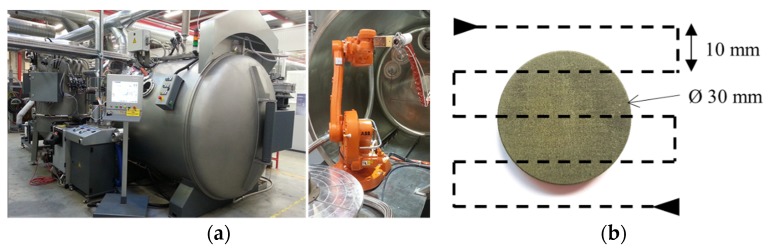
(**a**) Vacuum plasma spray system; (**b**) C/C composite substrate and robot path.

Figure 1a shows a 55 kW VPS system (Oerikon Metco, Wolhen, Switzerland), including a plasma spray gun (F4VB, Metco, Wolhen, Switzerland), which was mounted on a six-axis robot. The VPS coatings were applied to the substrate following the serpentine pattern shown in Figure 1b, which was controlled using the robot. The HfC and TiC powders were sieved using sieve shakers (BA 200N, CISA, Barcelona, Spain) before use. A 635-mesh sieve was used for HfC to achieve a D50 particle size of 8.7 µm, while 325- and 500-mesh sieves were used for TiC to give a D50 of 17.7 µm.

**Figure 2:**
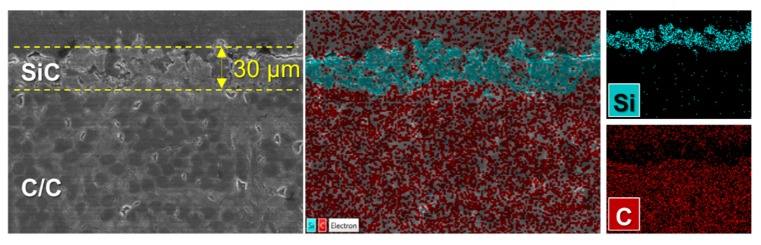
Cross-sectional field emission-scanning electron microscope (FE-SEM) image and energy dispersive spectrometer (EDS) analysis results of the substrate.

The substrates were C/C composites (Dai-Yang Industry Co., Icheon, Korea) covered with a silicon carbide (SiC) layer, as shown in Figure 2. The C/C composite of the substrate was manufactured by the needle punching method, and the SiC layer on the C/C composite was formed by the CVR method, in which Si in the SiO_2_ powder chemically reacts with the C/C composite surface to form SiC. The density of the substrate was 1.89 g/cm^3^, and the average of the surface roughness measured ten times was 3.79 µm. The SiC layer prevents the C/C composite surface from being damaged by the high-speed particles during the coating process, thereby forming a high quality UHTC coating layer. Foreign substances were removed from the surface of the substrate by ultrasonic cleaning prior to the coating process to improve the adhesion between the coating layer and the substrate.

**Figure 3:**
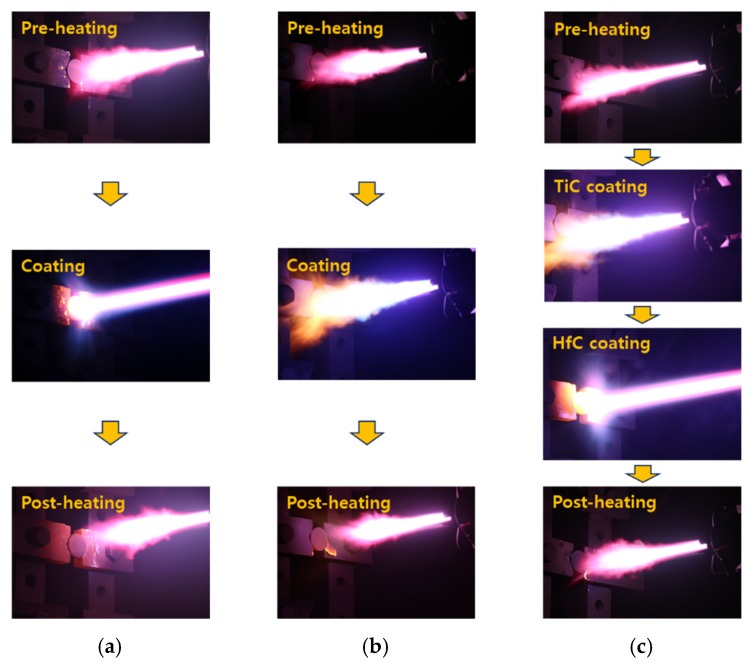
Plasma spray coating process. (**a**) HfC coating; (**b**) TiC coating; (**c**) HfC/TiC coating.

Figure 3 shows the plasma spray coating process of single-layer HfC and TiC coatings and multilayer HfC/TiC coatings. The process included pre-heating, coating, and post-heating steps, where pre-heating and post-heating were performed to prevent cracking and delamination of the coating layer caused by differences in the thermal expansion coefficients between the substrate and coating layer [14]. For the multilayer HfC/TiC samples, the layers were coated sequentially in the vacuum chamber. The shape of the plasma flame shown in the images changed depending on the processing conditions, such as the pressure, gas type and flow rate, and spray distance.

Table 1 shows the VPS conditions for the single-layer HfC and TiC coatings and multilayer HfC/TiC coatings on the C/C composite substrates. Since the melting point of HfC is approximately 3900 °C, the powder used in the coating process was chosen to be of a small size. Therefore, it must be sprayed at high velocity at a low pressure of 50 mbar and be sufficiently melted while spraying for a distance of 350 mm. The melting point of TiC is 3160 °C, and it is sufficiently melted even with a current of 720 A. The coating process was carried out at a pressure of 200 mbar for the coating rate. After coating, the specimen was slowly cooled in the vacuum chamber under an argon atmosphere to below 100 °C. Then, the samples were removed from the chamber and measurement specimens were fabricated using a diamond wheel cutter (Brillant 220, ATM, Mammelzen, Germany).

Cross-sectional images of the samples were obtained using FE-SEM (SU8030, Hitachi, Tokyo, Japan), and the composition of the coating layer was analyzed using EDS (X-MaxN80, Horiba, Kyoto, Japan). The porosity was measured from optical microscopy (Eclipse LV 100P OL, Nikon, Tokyo, Japan) images using image analysis software (NeoMeasure v2.0, ITechOne, Daejeon, Korea) following ASTM: E-2109 (Test Methods for Determining Area Percentage Porosity in Thermal Sprayed Coatings). The hardness of the coated layer was measured using a Vickers hardness tester (HM-210, Mitutoyo, Tokyo, Japan), while the surface roughness was measured using a non-contact 3D surface measurement system (IFM G4, Alicona, Graz, Austria).

## 3. Results and Discussion

**Figure 4:**
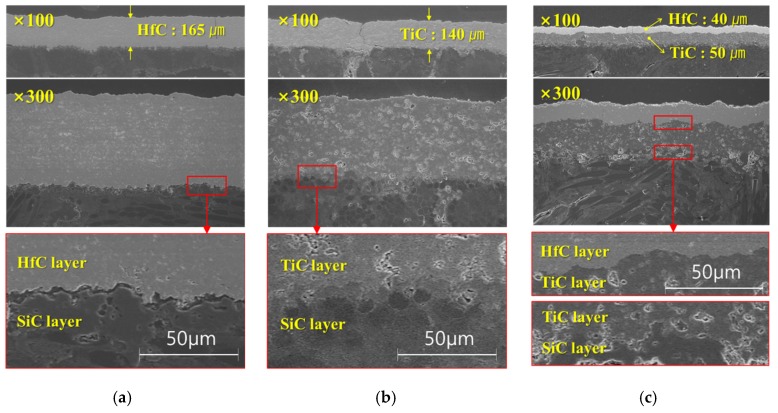
FE-SEM images of (**a**) HfC coating, (**b**) TiC coating, and (**c**) HfC/TiC coatings.

The FE-SEM images of the sample cross sections are shown in Figure 4. The coating thicknesses of the HfC and TiC single-layer coatings were 165 µm and 140 µm, respectively, while the thicknesses of the HfC and TiC layers in the HfC/TiC multi-layer coating were 40 µm and 50 µm, respectively. The layer thickness can be determined by the powder feed rate and the number of coating cycles. If the thickness of the coating layer is increased, the integrity of the coating layer is lowered due to the brittle characteristic of the ceramic. Therefore, in this study, the coating was applied to the thickness of which the integrity was confirmed. It can be seen for the HfC sample that the coating layer was not well-bonded to the substrate. However, the bonding between the TiC coating and the substrate was excellent. Hence, we formed the HfC/TiC multilayer structure with TiC in contact with the substrate to take advantage of the excellent thermal characteristics of HfC and the excellent bonding behavior of TiC (which was confirmed by the SEM image shown in Figure 4c). 

**Figure 5:**
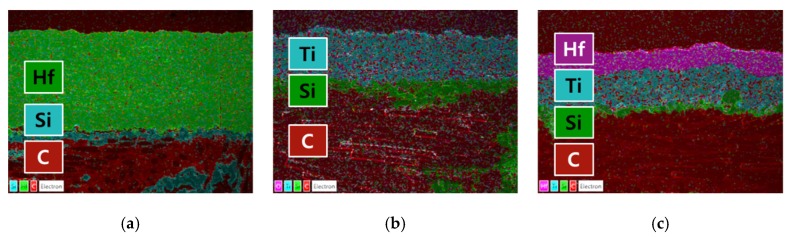
EDS mappings of (**a**) HfC coating, (**b**) TiC coating, and (**c**) HfC/TiC coatings.

Figure 5 shows the EDS results for the coated layers in the form of elemental maps. No oxides were observed in any of the coating layers. Prior to VPS coating, the pressure was maintained at 0.5 mbar in the vacuum chamber and then the chamber was filled with Ar at 30 mbar. The production of oxides was further suppressed since a reducing gas mixture of Ar/H_2_ was used as the processing gas at 50–200 mbar.

**Figure 6:**
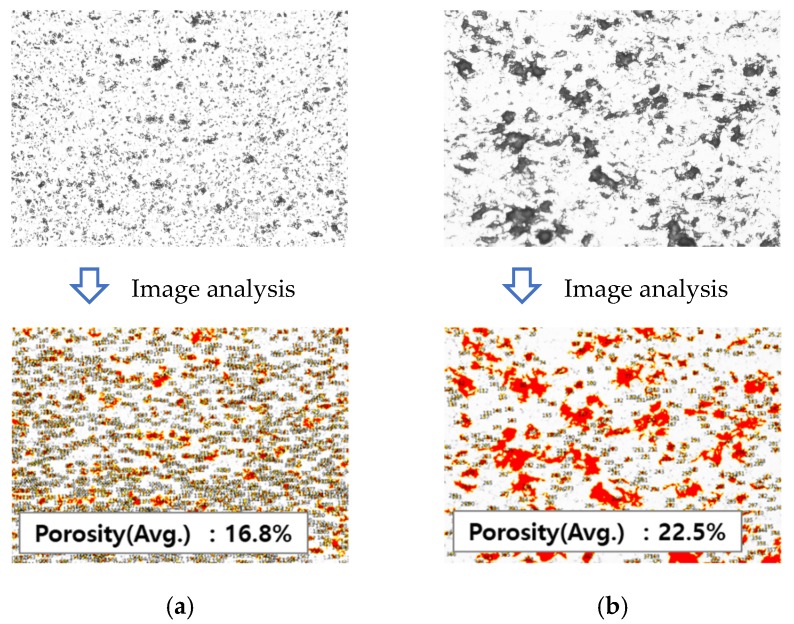
Porosity measurements of the (**a**) HfC and (**b**) TiC single-layer coatings. The upper images show optical microscopy images, while the lower images show the pores identified using the image analysis software.

The process for measuring the porosity from optical microscopy using image analysis software is shown in Figure 6. Five random areas for each coating layer specimen were analyzed, and average porosity values of approximately 16.8% for the HfC coating and 22.5% for the TiC coating were determined. The HfC coating showed a lower porosity and smaller pore size than the TiC coating, which were attributed to the lower pressure used during coating. When the processing pressure decreases, the particle velocity increases and the impact force of the droplets of melted powder hitting the substrate increases, resulting in a high-density coating being formed. The porosity is also related to the size of the powder. The HfC powder size is 8.7 µm and the TiC powder size is 17.7 µm. As the size of the powder increases, the porosity increases because the pore size between particles and particles increases when coated on the substrate.

**Figure 7:**
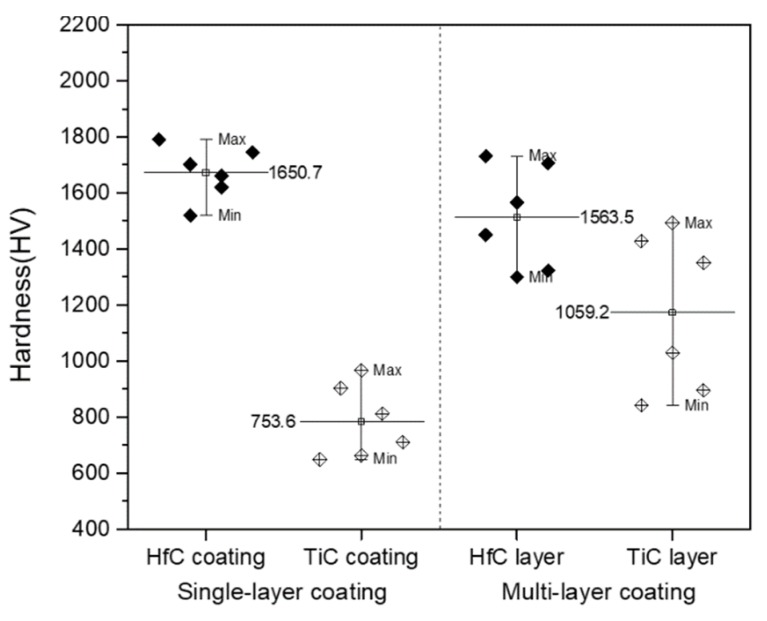
Vickers hardness of the HfC, TiC, and HfC/TiC coatings.

Figure 7 shows the Vickers hardness of the three different coating layers. The hardness of the cross sections was measured using a Vickers hardness tester with a 300 gf test force for 14 s. The Vickers hardness was measured twelve times in total, where the maximum and minimum values were excluded and the value shown is the average of the remaining ten values. The hardness values of the HfC and TiC coatings were 1650.7 HV and 753.6 HV, respectively. The hardness values of the HfC and TiC layers in the multilayer sample were 1563.5 HV and 1059.2 HV, respectively. Because the multilayer coating had layer thicknesses of 40–50 µm, relatively large measurement errors were obtained, resulting in a larger range of values than observed for the single-layer coatings. In the case of HfC, similar values for both the single and multilayer coatings were observed, while for TiC, the hardness was higher in the multilayer coating. This was attributed to the decrease in the feeding rate, from 4.5 g/min for the single layer to 2 g/min for the multilayer, while the coating cycle was two in both cases. When the coating cycle increases, the error in the process decreases, and the uniformity of the coating over the substrate area improves. When preparing the TiC coating for the multilayer, the coating cycle was maintained at two. However, the feeding rate was reduced to achieve a coating thickness of ~50 µm, which resulted in the higher Vickers hardness compared to the single TiC coating. The hardness is related to the porosity. The high porosity shows the low density of the coating layer and therefore the hardness becomes low. In addition, since the pore size of the TiC coating layer is larger than the pore size of the HfC coating layer, it shows the low hardness.

**Figure 8:**
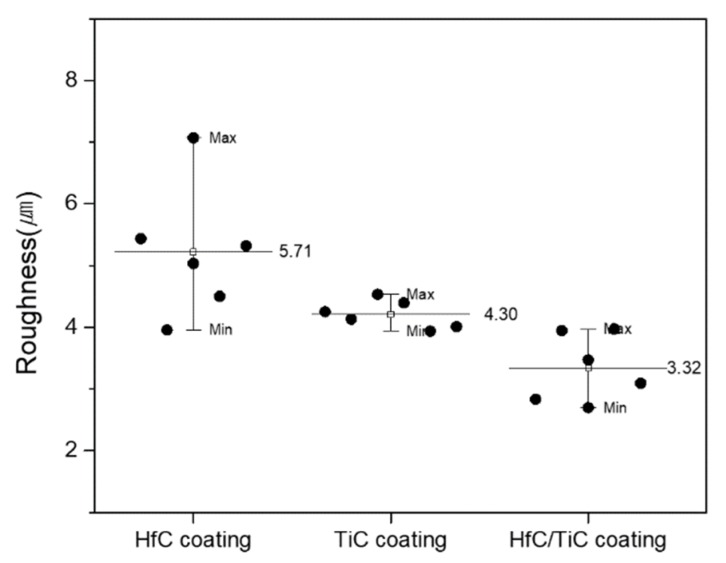
Surface roughness values of the HfC, TiC, and HfC/TiC coatings.

**Figure 9:**
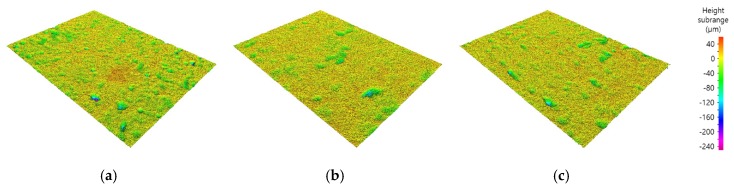
Surface texture of (**a**) HfC, (**b**) TiC, and (**c**) HfC/TiC coatings.

The surface roughness of each coating was measured using a non-contact 3D surface measurement system. The roughness was measured twelve times over a length of 5 mm for each measurement, where the values shown in Figure 8 are the average of ten values (excluding the maximum and minimum values). The roughness values were 5.71 µm for the HfC coating, 4.30 µm for the TiC coating, and 3.32 µm for the HfC/TiC coating. Although the HfC coatings on the surface of both the single and multilayer specimens were prepared using the same processing conditions, the HfC/TiC multilayer coating had a better roughness. This was confirmed by the better characteristics of the TiC coating compared with the HfC coating for single-layer coatings. As the melting point of TiC is relatively low, the amount of unmelted powder inside the plasma flame is relatively small and the coating rate and coating surface state are good. The TiC layer with such characteristics acted as a bonding layer between the substrate and HfC layer in the multilayer coating, thereby contributing to the better adhesion and lower surface roughness. Figure 9 shows the surface texture of each specimen measured using the non-contact 3D surface measurement system, which allows the state of the coating surface to be easily verified visually. In the case of the HfC/TiC multilayer coating, although the surface HfC layer was identical to that of the single HfC coating, the surface of the multilayer was more uniform due to the smoother underlying TiC layer.

## 4. Conclusions

To prevent the oxidation of C/C composites at high temperatures, we applied single-layer HfC and TiC coatings and HfC/TiC multilayer coatings using a 55 kW VPS system. The coating thickness of each specimen was examined using FE-SEM, and excellent bonding between the coating and substrate was confirmed for the HfC/TiC multilayer coating. The powder sizes of HfC and TiC were 8.7 µm and 17.7 µm, respectively. When the molten powders of TiC were laminated to the substrate in splat form, they were bonded in a larger area than HfC. For this reason, the bonding state between TiC and the substrate is excellent. The composition of the coating layer was analyzed using EDS, which confirmed that no oxides were formed. This is because the VPS coating process is performed in a vacuum chamber in an Ar atmosphere. The porosity of the coating layer was analyzed using microscopy images, which indicated that the HfC coating had sufficiently high density. The Vickers hardness of each coating layer was compared. HfC is performed under the pressure of 50 mbar, and the acceleration speed of the powder is faster than that of TiC. For this reason, the porosity of HfC is superior to that of TiC. It can be confirmed that the Vickers hardness is also excellent. The surface roughness of the coating was analyzed using a non-contact 3D surface measurement system, where the effect of the TiC layer as the interlayer in the multilayer coating was investigated. Furthermore, to visually confirm the surface quality, images of the surface textures were investigated. Our future work is an ablation test on ultra-high temperature ceramic coatings made for the purpose of oxidation prevention. The ablation test will be carried out using a plasma wind tunnel and evaluated through mass loss and recession before and after the experiment.

## Figures and Tables

**Table 1:** Experimental conditions for the plasma spray coating.

Condition	HfC Coating	TiC Coating	HfC/TiC Coating	
			HfC Layer	TiC Layer
Current (A)	780	720	780	720
Pressure (mbar)	50	200	50	200
Ar gas flow (L/min)	35	35	35	35
H_2_ gas flow (L/min)	10	12	10	12
Carrier gas flow (L/min)	1.6	2	1.6	2
Spray distance (mm)	350	220	350	220
Feed rate (g/min)	8	4.5	8	2
Coating cycle (no.)	30	2	6	2

## Nomenclature

CVR	Chemical vapor reaction
EDS	Energy dispersive X-ray spectroscopy
FE-SEM	Field-emission scanning electron microscopy
UHTC	Ultra-high-temperature ceramic
UTM	Universal testing machine
VPS	Vacuum plasma spraying
